# Opposing effects of Toll-like receptors 2 and 4 on synaptic stability in the spinal cord after peripheral nerve injury

**DOI:** 10.1186/1742-2094-9-240

**Published:** 2012-10-23

**Authors:** Camila Marques Freria, Licio Augusto Velloso, Alexandre LR Oliveira

**Affiliations:** 1 Department of Structural and Functional Biology, Institute of Biology, University of Campinas (UNICAMP), CP 6109, CEP 13083–970, Campinas, SP, Brazil; 2 School of Medical Sciences, University of Campinas (UNICAMP), Campinas, SP, Brazil

## Abstract

**Background:**

Glial cells are involved in the synaptic elimination process that follows neuronal lesions, and are also responsible for mediating the interaction between the nervous and immune systems. Neurons and glial cells express Toll-like receptors (TLRs), which may affect the plasticity of the central nervous system (CNS). Because TLRs might also have non-immune functions in spinal-cord injury (SCI), we aimed to investigate the influence of TLR2 and TLR4 on synaptic plasticity and glial reactivity after peripheral nerve axotomy.

**Methods:**

The lumbar spinal cords of C3H/HePas wild-type (WT) mice, C3H/HeJ TLR4-mutant mice, C57BL/6J WT mice, and C57BL/6J TLR2 knockout (KO) mice were studied after unilateral sciatic nerve transection. The mice were killed via intracardiac perfusion, and the spinal cord was processed for immunohistochemistry, transmission electron microscopy (TEM), western blotting, cell culture, and reverse transcriptase PCR. Primary cultures of astrocytes from newborn mice were established to study the astrocyte response in the absence of TLR2 and the deficiency of TLR4 expression.

**Results:**

The results showed that TLR4 and TLR2 expression in the CNS may have opposite effects on the stability of presynaptic terminals in the spinal cord. First, TLR4 contributed to synaptic preservation of terminals in apposition to lesioned motor neurons after peripheral injury, regardless of major histocompatibility complex class I (MHC I) expression. In addition, in the presence of TLR4, there was upregulation of glial cell-derived neurotrophic factor and downregulation of interleukin-6, but no morphological differences in glial reactivity were seen. By contrast, TLR2 expression led to greater synaptic loss, correlating with increased astrogliosis and upregulation of pro-inflammatory interleukins. Moreover, the absence of TLR2 resulted in the upregulation of neurotrophic factors and MHC I expression.

**Conclusion:**

TLR4 and TLR2 in the CNS may have opposite effects on the stability of presynaptic terminals in the spinal cord and in astroglial reactions, indicating possible roles for these proteins in neuronal and glial responses to injury.

## Introduction

Peripheral nerve lesions lead to local and retrograde inflammation, resulting in synaptic changes in the central nervous system (CNS). The mechanisms that trigger such changes are not fully understood, but it is clear that molecules classically related to the immune system are pivotal. Expression of major histocompatibility complex class I (MHC I) by neurons and glial cells has been implicated in the synaptic elimination process during development and after lesions in adulthood [1-3]. More recently, molecules from the classic complement pathway have also been implicated in the process of refinement of neural circuits and as important players in the response to peripheral nerve injury [4]. This classic model for studying the retrograde reaction to axon transection has been widely used, and has improved our understanding of the mechanisms underlying synapse elimination and of the interactions between neurons and glial cells after injury [2,5,6].

Another class of innate immune-system molecules that may play a role in synaptic plasticity are the Toll-like receptors (TLRs). TLRs are transmembrane proteins that play crucial roles as pattern-recognition receptors. They are expressed by macrophages, microglia [7-10], astrocytes [10,11], Schwann cells [12], and neurons [13]. TLRs contribute to the initial induction of neuroinflammation in the CNS, which is predominantly modulated by microglia and astrocytes [9,10,14,15]. Both i*n vivo* and *in vitro* studies show that TLR4 is mostly expressed by microglia [7,8], whereas TLR2 can be expressed by microglia [10] and astrocytes [14,16].

In addition to their involvement with the neuronal response to injury, glial cells have been implicated in remodeling of the CNS after lesion. Peripheral nerve sectioning in adult animals results in astrogliosis and microglial reaction in the surroundings of the axotomized motor neuron cell bodies within the ventral horn of the spinal cord [5].

Of particular interest in this model is the process of synaptic elimination that takes place in the surroundings of the α-motor neurons. Based on ultrastructural observations, parts of the inhibitory synapses containing glycine and γ-aminobutyric acid (GABA) are selectively kept in apposition to the lesioned motor neurons. By contrast, most of the excitatory presynaptic terminals undergo retraction by the interposition of glial processes between the terminals and the postsynaptic membrane [17].

There is evidence that both the degree of glial reaction and the type of surface molecules that are expressed by glial cells may influence the stability of neural circuits and the regenerative outcome [2,5,18,19]. Taking into account the profound changes in spinal-cord circuits that occurs after a peripheral lesion, it is possible that TLRs are involved in signaling pathways that also involve glia and neurons. As a consequence, TLRs might be involved in synaptic stability either directly or by modulating glial reactivity.

To confirm these possibilities, we investigated the process of synaptic plasticity after peripheral axotomy in the spinal cord of mutant mice with absent or non-functional TLR2 or TLR4. We also analyzed astroglial reactivity *in vitro*. We found that TLR2 and TLR4 have opposite functions in the spinal-cord microenvironment. TLR4 contributes to the preservation of synaptic terminals in apposition to lesioned motor neurons after peripheral injury, regardless of glial reactivity, whereas TLR2 leads to synaptic preservation and upregulation of MHC I expression, thereby downregulating astroglial reactivity.

## Methods

### In vivo *experiments*

#### Ethics approval

The institutional committee for ethics in animal experimentation approved the study (CEUA/IB/UNICAMP, proc. 1656–1), and all housing, surgical, and postoperative care procedures were performed in accordance with the guidelines of the Brazilian National Council for Control of Animal Experimentation (CONCEA).

#### Animals

Adult male mice (6–8 weeks old) of the following strains were obtained from the Multidisciplinary Center for Biological Investigation (CEMIB/UNICAMP): C3H/HePas (wild-type (WT), n = 20), C3H/HeJ (TLR4 mutant, n = 20), C57BL/6J (WT, n = 20) and C57BL/6J (TLR2^−/−^ KO mice, n = 20). The C3H/HeJ mutant mice, which express a non-functional TLR4 protein, have a point mutation in the receptor’s cytosolic domain [20].

#### Surgical procedures and tissue preparation

All mice were anaesthetized with a mixture of xylasine 10 mg/kg (Kensol, Köning, Avellaneda, Argentina) and ketamine 50 mg/kg (Vetaset, Fort Dodge Laboratories, IA, USA) at a proportion of 1:1, given as intraperitoneal injection of 0.12 mL/25 g), followed by transection of the left sciatic nerve. A segment 1 mm in length of the distal stump was removed, to avoid direct contact between the stumps. The muscle and skin layers were sutured, and the animals kept in the vivarium of the Laboratory of Nerve Regeneration (UNICAMP) for 1 week.

At this time (1 week after axotomy), all animals were killed with an overdose of anesthetic, then *trans*-cardiac perfusion was performed with 0.1 mol/l PBS (20 ml, pH 7.4). Tissue was fixed with 10% formaldehyde in PBS for immunohistochemistry or with 2.5% glutaraldehyde and 1.0% paraformaldehyde in phosphate buffer 0.1 mol/l (pH 7.4) for transmission electron microscopy (TEM). The lumbar enlargements (L4 to L6) of the spinal cords were removed, cryoprotected by immersing in 30% sucrose in phosphate buffer 0.1 mol/l for 12 hours, and either frozen (immunohistochemistry) or embedded in resin (TEM).

#### Immunohistochemistry

The lumbar spinal cords were frozen in isopentane at −40°C for sectioning on a cryostat sectioning at 12 μm. The sections were transferred to gelatin-coated slides and blocked in Tris-buffered saline with Triton X-100 (TBS-T) with 3% BSA at room temperature for 1 hour. The spinal-cord sections from axotomized mice were incubated overnight at 4°C in a moist chamber with a rat anti-MHC I antibody at 1:100(T-2104 for C3H/HeJ and C3H/HePas mice and T-2105 for C57BL/6J and TLR2 KO mice; both Peninsula Laboratories, San Carlos, CA, USA), rabbit anti-synaptophysin (1:100; Dako, Carpinteria, CA, USA), goat anti-glial fibrillary acidic protein (GFAP; 1:200; Santa Cruz Biotechnology, Santa Cruz, CA, USA) and rabbit anti-Iba1 (1:700; Wako Chemicals USA, Richmond, VA, USA), diluted in TBS-T with 1% BSA. After a further set of washes in TBS-T, the sections were incubated with Cy3-conjugated or Cy2-conjugated secondary antibodies (1:250; Jackson ImmunoResearch, Bar Harbor, ME, USA) for 1 hour in a moist chamber at room temperature. The slides were then rinsed in TBS-T, mounted in a mix of glycerol and PBS (3:1 ratio), and viewed under a fluorescence microscope (Eclipse TS100, Nikon, Tokyo, Japan) equipped with a digital camera (DXM1200F; Nikon, Tokyo, Japan), which recorded photographs.

For quantitative measurements, three alternate sections from the same level of the spinal cord (ipsilateral and contralateral sides of the spinal cord) from each animal (*n* = 5 for each group) were used to capture images from the ventral horn at a final magnification of × 200, always using identical settings. Double-blind assessment of the data was not possible because the mutant and KO mice could not be obtained concurrently, owing to breeding peculiarities. Quantification was performed with the enhance contrast and density slicing feature of ImageJ software (version 1.33; National Institutes of Health, Bethesda, MD, USA). In this way, the threshold of each image was manually obtained by comparison with the initially captured images. The integrated density of pixels was systematically measured in six representative areas around each motor neuron (dorsolateral lamina IX, lesioned and unlesioned sides) using a circular sampling area of ~80 μm^2^, as described previously [2]. The regions of interest were equally distributed along the membrane cell body of the motor neuron, as exemplified in (see Additional file 1: Figure S14D). The ipsilateral:contralateral ratio of the integrated density of pixels was calculated for each section and expressed as the mean value for each spinal-cord sample. The data are represented as the mean ± standard error of the mean (SEM). A summary of the immunohistochemistry quantification procedure is available as a supplementary material (see Additional file 1: Figure S14).

#### Electron microscopy

After fixation, the spinal cords of lesioned mice (n = 5 for each group) were treated with osmium tetroxide, dehydrated, and embedded in epoxy resin (Durcupan ACS; Fluka, Steinheim, Switzerland). Ultrathin cross-sections obtained from the lumbar enlargement were collected on formvar-coated copper grids, contrasted with uranyl acetate and lead citrate, and examined under a TEM (Tecnai G^2^ Spirit BioTWIN; FEI Company, Eindhoven, the Netherlands) operated at 120 kV.

Neurons with large cell bodies (>35 μm in diameter), which were found in the sciatic motor-neuron pool and cut in the nuclear plane, were identified as α-motor neurons by the presence of C-type nerve terminals. The surface of the cells was then sequentially captured with a digital camera at a magnification of × 11,000, and the images were mounted together using vectorial software. Boutons were classified into three different synaptic types (F, S and C terminals), following the nomenclature of Conradi [21]. Type S terminals, with spherical vesicles and asymmetrical active zone, contain the excitatory neurotransmitter glutamate‘ C terminals contain spherical vesicles loaded with acetylcholine, and present a characteristic subsynaptic cistern; and F terminals contain flattened or pleomorphic vesicles filled with glycine and/or γ-aminobutyric acid (GABA) and function as inhibitory inputs. The relatively infrequent glutamatergic M-boutons, containing spherical synaptic vesicles and originating from Ia primary afferent fibers, were typed as S-boutons. For each neuron, we calculated the number of synaptic terminals per 100 μm of cell membrane, and the percentage membrane length, using the measurement tool of the ImageTool software (version 3.0; University of Texas Health Center, San Antonio, TX, USA). A total of 80 motor neurons were studied (four neurons per animal, n = 5 per group) for the four mouse genotypes. Double-blind assessment of the data was not possible, because the mutant and KO mice could not be obtained concurrently owing to breeding particularities.

#### Western blotting

To quantify levels of synaptophysin and MHC I, samples (3 mm) of the lumbar spinal cords (both lesioned and unlesioned sides) were excised. The specimens then underwent sonication for 1 minute in RIPA protein extraction buffer (150 mmol/l NaCl, 50 mmol/l Tris pH 8.0, 1 mmol/l phenylmethanesulfonylfluoride, 1 mmol/l EDTA, 0.5% Na-deoxycholate acid, 0.1% SDS and 1% Triton X-100). The total protein concentration was measured using the Bradford protein assay (Bio-Rad Laboratories, Inc., Hercules, CA, USA), and the protein samples used for western blotting. Aliquots (40–60 μg) of protein for each tissue sample were separated in a 10% polyacrylamide gel under reducing conditions. The proteins were electrically transferred to nitrocellulose membranes (Hybond-ECL; Amersham Biosciences, Chalfont St. Giles, UK), which were blocked at room temperature with shaking for 1 hour with TBS-T containing 5% non-fat dry milk. Rat anti-MHC class I (MHC I T2104 and T2105 monoclonal antibodies (Peninsula), diluted 1:1000 in TBS-T containing 1% non-fat dry milk in TBS-T) and rabbit anti-synaptophysin (monoclonal antibody (Dako), diluted 1:500 in TBS-T containing 5% non-fat dry milk) were incubated overnight at 4°C. Three TBS-T washes were carried out, and then horseradish peroxidase-conjugated rabbit anti-rat IgG and goat anti-rabbit IgG, both diluted 1:2000 in TBS-T (both Zymed Laboratories, San Francisco, CA, USA) were added for 1 hour at room temperature with shaking. After another set of washes, the bound antigens were detected by chemiluminescence (Perkin-Elmer, Waltham, MA, USA). Band intensity was determined by densitometry using ImageJ software. Loading control experiments were performed with anti-rabbit β-actin polyclonal antibody 1:1000 (Abcam, Cambridge, MA, USA).

#### Reverse transcriptase PCR

Relative mRNA levels of β2-microglobulin, intereukin (IL)-6, IL-1β, brain-derived neurotrophic factor (BDNF) and glial cell-derived neurotrophic factor (GDNF) were measured in the right and left sides of the lumbar spinal cords of C57BL/6J (WT) and TLR2^−/−^ (KO), C3H/HePas (WT), and C3H/HeJ (mutant) mice after left sciatic nerve axotomy.

The samples were placed in a sonicator for 30 seconds, and then total RNA was extracted (RiboZol reagent; Amresco, Solon, OH, USA), in accordance with the manufacturer’s instructions. The obtained RNA was purified using a commercial kit (RNeasy Mini Kit; Qiagen Inc., Valencia, CA, USA). Each sample (1 μg) of RNA was reverse-transcribed using a commercial kit (AffinityScripts QPCR cDNA Synthesis Kit; Agilent Technologies, La Jolla, CA, USA) in a final reaction volume of 20 μL. Real-time quantitative PCR was performed using a SYBR Green real-time PCR kit [on a quantitative PCR system (Mx3005P QPCR System; Agilent Technologies), with an initial denaturation for 10 minutes at 95°C, followed by 45 cycles of amplification (95°C for 30 seconds followed by 72°C for 1 minute). The reactions were carried out with 12.5 μl 2 × SYBR Green PCR master mix (Agilent Technologies), 0.2 μmol/l of each forward and reverse primer, and 50 ng cDNA template in a final reaction volume of 20 μl. Melting curve analyses were performed at the end of the PCR to verify the identities of the products. Melting curves occurred at 95°C for 60 seconds and 55°C for 30 seconds.

All quantifications were normalized to the housekeeping gene glyceraldehyde 3-phosphate dehydrogenase. A non-template control with no genetic material was included to control for contamination and nonspecific reactions. Each sample (n = 4) was tested in triplicate and then used for the analysis of the relative transcription data using the 2^−ΔΔCT^ method (Livak and Schmittgen, 2001). The data are expressed as the lesioned/unlesioned ratio, adopting the unlesioned side of each genotype as 100%. The primer sequences are available as supplementary material (Table 1).

**Table 1:** Primer sequences used for real-time reverse transcriptase PCR

**Primer**		**Sequence5**^**′**^**→3**^**′**^
β2-microglobulin	Forward	ATGGCTCGCTCGGTGACCCTG
	Reverse	CCGGTGGGTGGCGTGAGTATACTT
GAPDH	Forward	TGCACCACCAACTGCTTA
	Reverse	GGATGCAGGGATGATGTTC
IL-6	Forward	AGTGGCTAAGGACCAAGACCATCCA
	Reverse	5^′^GGCATAACGCACTAGGTTTGCCGA
IL-1β	Forward	5^′^GAGCTTGACGGCACCCTCGC
	Reverse	AGCTTCGTGGCTGTGGAAAAAGTGT
BDNF	Forward	5^′^ CACTCCGACCCTGCCCGC
	Reverse	CCCGCCAGACATGTCCAC
GDNF	Forward	5^′^ TGCCCGCCGGTAAGAGGCTT
	Reverse	TGGAGTCACTGGTCAGCGCGAA

BDNF, brain-derived neurotrophic factor; GADPH, glyceraldehyde 3-phosphate dehydrogenase; GDNF, glial cell-derived neurotrophic factor; IL, interleukin.

### In vitro *experiments*

#### Cell culture

Primary cultures of astrocytes were prepared as described previously [22] from the cerebral cortices of C3H/HePas (n = 5), C3H/HeJ (n = 5), C57BL/6J (n = 5) and TLR2^−/−^ KO mice (n = 5) (all from CEMIB/UNICAMP) at 1 to 2 days old. Briefly, the cortical hemispheres from neonatal mice were excised and, after removal of the meninges and blood vessels, were chopped and incubated in 0.05% trypsin in PBS for 10 minutes. DNase was added to the pre-digested tissue, and the resulting cell suspension in 4% bovine serum albumin in Dulbecco’s modified Eagle’s medium was spun in a centrifuge for 10 minutes at 250 ×g. The cell precipitate was re-suspended in DMEM supplemented with 10% fetal bovine serum (FBS), penicillin and streptomycin (1 μl/ml,), glucose (16 μl/ml) (all Nutricell, Campinas, SP, Brazil), nerve growth factor (NGF; 0.25 μl/ml), and insulin (1 μl/ml) (both Sigma-Aldrich, St Louis, MO, USA), and then seeded into cell culture flasks (25 cm^2^). The resulting primary astrocyte cultures were kept in an incubator at 37°C in 95% O_2_/5% CO_2_ for 1 week. Upon confluence, the cultures were treated with trypsin again and spun for 10 minutes I a centrifuge. The pellet was re-suspended in glial medium (GM) and seeded at 2.5 × 10^4^ cells/well in 24-well culture plates (Corning/Costar Corp., Cambridge, MA, USA), which were placed in an incubator under the same conditions (37°C, 95% O_2_/5% CO_2_). The GM was renewed every other day, and all experiments were performed in triplicate.

#### Analysis of cell proliferation

After 24 hours, 30 μmol/l of 5-bromo-2^′^-deoxyuridine (BrdU, Sigma-Aldrich) was added to the cultured cells in 24-well plates. Three wells per group were fixed with 4% paraformaldehyde (Reagen, Rio de Janeiro, RJ, Brazil) in PBS. The other wells were fixed with the same procedure after 2 and 3 days of culturing, respectively. The fixed cultures were incubated in blocking buffer (PBS containing 0.1% Tween and 5% BSA) for 1 hour at room temperature. After blocking, cells were incubated at 37°C for 80 minutes with anti-BrdU (1:400; Abcam) in blocking buffer containing 5 mmol/l MgCl_2_ and 0.5 μl/ml DNase. After a further set of washes in PBS plus 1% Tween 20, the cells were incubated in Cy3-conjugated secondary antibody (1:250, Jackson ImmunoResearch Laboratories Inc., West Grove, PA, USA) for 45 minutes at room temperature. Nuclei were labeled with 4^′^,6-diamidino-2-phenylindole (DAPI; Dako) for 10 minutes. The cultures were rinsed in 0.1 mol PBS, mounted in a mixture of glycerol:PBS (3:1) and examined under an inverted microscope (Eclipse TS100; Nikon, Tokyo, Japan) connected to a camera (DXM1200F; Nikon). The number of astrocytes was determined with ImageTool by counting the DAPI-stained nuclei. The mitotic rate was calculated from the ratio of BrdU/DAPI labeling in 12 randomly obtained areas, documented for each day of culture fixation. Three points (24, 48 and 72 hours) were obtained from the mean value calculated for each group studied.

#### Immunocytochemistry

At 1 week after culturing, the astrocytes were fixed with 4% paraformaldehyde in PBS, rinsed several times in PBS and incubated in TBS-T with 3% BSA at room temperature for 1 hour. The cultures were further incubated for 2 hours with the primary antibody goat anti-GFAP (1:100; Santa Cruz) diluted in TBS-T containign 1% BSA. Next, the cultures were rinsed in TBS-T and incubated for 45 minutes with Cy3-conjugated secondary antisera (1:250; Jackson ImmunoResearch Laboratories). Nuclei were labeled with DAPI for 10 minutes and rinsed with PBS. The preparations were then mounted in a mixture of glycerol:PBS (3:1), examined under an inverted microscope (Eclipse TS100; Nikon) and quantified using ImageJ. To analyze GFAP labeling, the integrated density of pixels was measured at six random areas in each well (four wells in total). The average integrated density of pixels was calculated for each well and then for each group, and compared between them. The average labeling was normalized per 1x10^5^ μm^2^ of surface area. The data are represented as the mean ± SEM.

#### Statistical analysis

The data are presented as the mean ± SEM, and the differences between groups were considered significant at P-value <0.05. Data were analyzed ANOVA followed by a Bonferroni *post hoc* test for parametric data or a Mann–Whitney *U*-test for non-parametric data.

## Results

### Toll-like receptors 2 and 4 have opposite effects on synaptic plasticity after peripheral nerve lesion

To evaluate the changes in synaptic covering after peripheral lesion, the spinal-cord sections were immunostained with anti-synaptophysin antiserum. Only large motor neurons present in the dorsolateral nucleus, which supplies the distal hind limb muscles, were considered for this analysis. The labeling found in the contralateral ventral horn was compared with the ipsilateral (lesioned) side.

**Figure 1:**
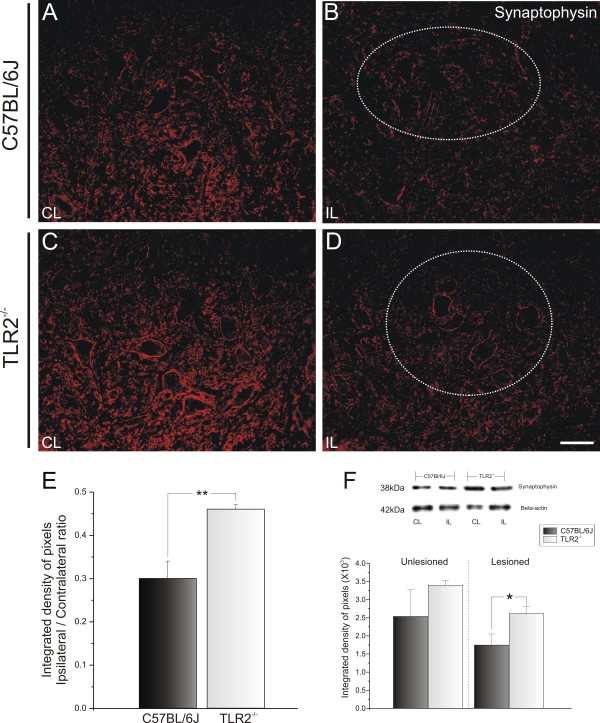
**Representative images of synaptophysin immunostaining in C57BL/6J wild-type (WT) and Toll-like receptor (TLR)2 knockout (KO) mice (TLR2**^**−/−**^**) 1 week after unilateral axotomy.** Note that 1 week after lesioning, there was a strong decrease in labeling, especially in the areas surrounding the motor neurons. This decrease was more intense in (**B**) C57BL/6J mice than (**D**) in TLR2^−/−^ mice. (**A**,**C**) Contralateral side of C57BL/6J WT and TLR2^−/−^ mice, respectively. The dashed circle indicates the motor nucleus containing the alpha motor neurons. (**E**) Graph representing the ipsilateral:contralateral (IL:CL) ratio of the integrated density of pixels ** *P*<0.01. (**F**) Western blot analysis of synaptophysin expression in WT and TLR2 KO mice. Note the significant preservation of synaptophysin in TLR2^−/−^ after peripheral axotomy. β-Actin was used as sample loading control. * *P*<0.05. Scale bar: 50 μm.

**Figure 2:**
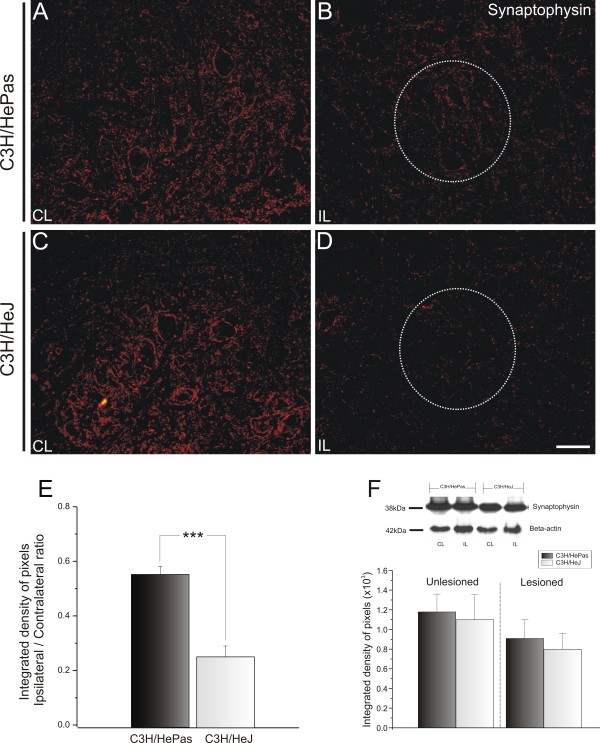
**Representative images of synaptophysin immunostaining in C3H/HePas WT and C3H/HeJ Toll-like receptor (TLR)4 mutant mice at 1 week after unilateral axotomy.** Note the stronger synaptic loss in (**D**) mutant mice compared with (**B**) WT. (**A** and **C**) Unlesioned side of WT and TLR4 mutant mice, respectively. The dashed circle indicates the motor nucleus containing the alpha motor neurons. (**E**) Graph representing the ipsilateral:contralateral (IL:CL) ratio of the integrated density of pixels. *** *P*<0.001. (**F**) Western blot analysis of synaptophysin expression in WT and TLR4 mutant mice. Note the similar expression of synaptophysin between strains. β-Actin was used as sample loading control. Scale bar: 50 μm.

Similar synaptophysin immunoreactivity was seen in the ventral horn of TLR2 and TLR4 mice (Figure 1; Figure 2A,C). In both cases, a clear decrease in synaptophysin labeling occurred in the motor nucleus on the lesioned side. However, the absence of TLR2 signaling led to a statistically significant greater preservation of the synaptic contacts. Thus, there was a significantly stronger reduction of the synaptic covering in C57BL/6J WT mice compared with TLR2^−/−^KO mice (0.30 ± 0.02 and 0.46 ± 0.01, respectively; ipsilateral:contralateral ratio, Student *t* test *P*<0.01) ( Figure 1E). However, the presence of TLR4 preserved the synaptic covering after lesion. There was greater synaptic preservation on the lesioned side of the C3H/HePas WT compared with that of the C3H/HeJ mutant mice (0.56 ± 0.03 and 0.26 ± 0.04, respectively; ipsilateral:contralateral ratio, Mann–Whitney *U*-test *P*<0.001 (Figure 2B,D; Figure 2

Similarly to the *in situ* immunolabeling results, western blotting analysis showed significant preservation of synapses on the lesioned side (contralateral: C57BL/6J 2.54 ± 0.73; TLR2^−/−^ 3.40 ± 0.13, Mann–Whitney *U* test *P*>0.05; ipsilateral: C57BL/6J 1.75 ± 0.3; TLR2^−/−^ 2.62 ± 0.20, Mann–Whitney *U* test *P*<0.05) (Figure 1F), but did not show significant differences between TLR4 mutant and WT mice (contralateral: C3H/HePas 1.18 ± 0.18; C3H/HeJ 1.10 ± 0.32, Mann–Whitney *U* test *P*>0.05; ipsilateral: C3H/HePas 0.92 ± 0.22; C3H/HeJ 0.80 ± 0.20, Mann–Whitney *U* test *P*>0.05) (Figure 2F).

**Figure 3:**
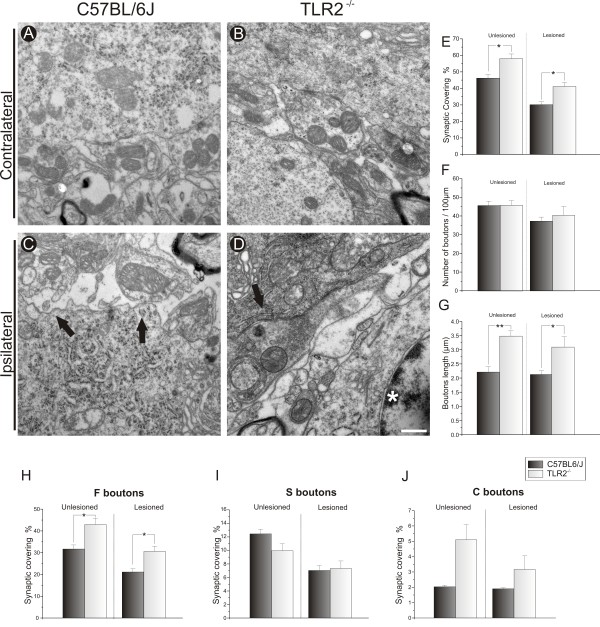
**Quantitative ultrastructural analyses of the number of synaptic terminals apposed to the surface of motor neurons after axotomy.** Representative micrographs of the surface of (**A**,**B**) contralateral and (**C**,**D**) ipsilateral motor neurons from C57BL/6J wild-type (WT) and TLR2^−/−^ mice strains. At1 week after peripheral lesion, (**C**) WT mice had greater detachment of synaptic terminals compared with (**D**) TLR2^−/−^ mice. The black arrows indicate the location of motor-neuron membrane surfaces from which synaptic terminals were (**C**) detached from or (**D**) in contact with the surface of the motor neuron, and the white asterisk indicates an astrocyte close to a synaptic terminal. (**E**) Graph of the total synaptic covering in unlesioned and lesioned neurons; there was greater synaptic covering in WT mice compared with TLR2^−/−^ mice on both sides. (**F**) Graph of the total number of synaptic terminals per 100 μm of motor-neuron membrane in lesioned and unlesioned neurons; note the absence of a difference between strains. (**G**) Graph of the bouton length on the motor-neuron membrane in lesioned and unlesioned neurons, showing differences before and after axotomy. (**H**-**J**) Graphs of F-, S- and C-terminal numbers per 100 μm of motor-neuron membranes, respectively; a greater loss of F-type terminals was seen 1 week after axotomy in WT mice. * *P*<0.05, ** *P*<0.01. Scale bar: 0,5 μm.

To detect the subtle changes that occur during synaptic elimination, a thorough ultrastructural analysis of the inputs apposed to α-motor neurons was carried out. The general ultrastructure appeared identical for all studied mice. The total synaptic covering, which represents the percentage of the motor-neuron body surface in contact with the presynaptic terminals, showed that 1 week after axotomy, the absence of TLR2 led to the preservation of synaptic covering, confirming the immunostaining results. Therefore, the mean total synaptic covering on both sides was greater in KO mice compared with WT mice (contralateral: C57BL/6J 46.24% ± 2.06%; TLR2^−/−^ 58.00% ± 2.81%.; Student *t* test *P*<0.05; ipsilateral: C57BL/6J 30.15% ± 1.73%; TLR2^−/−^ 41.12% ± 2.43%, Student *t* test *P*<0.05) (Figure 3E). However, the greater preservation on both sides was the result of greater bouton length (μm) rather than a greater number of terminals (mean length in μm: contralateral: C57BL/6J 2.22 ± 0.18, TLR2^−/−^ 3.49 ± 0.18, Student *t* test *P*<0.01; ipsilateral: C57BL/6J 2.13 ± 0.13; TLR2^−/−^, 3.10 ± 0.36, Student *t*-test *P*<0.05) (Figure 3G).

**Figure 4:**
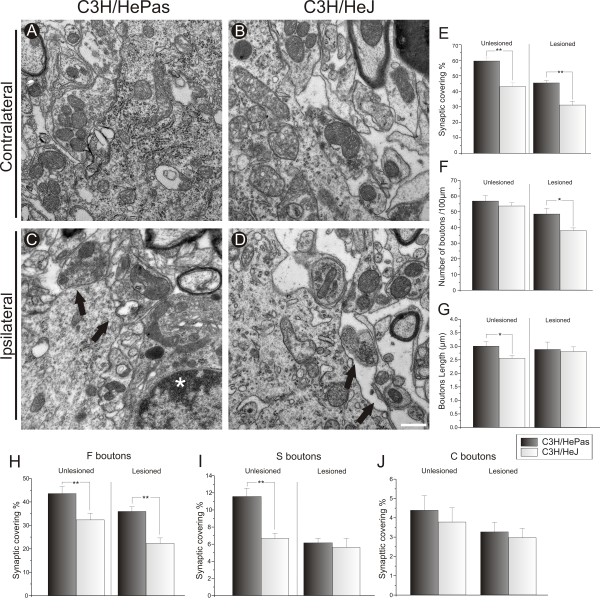
**Quantitative ultrastructural analyses of the number of synaptic terminals apposed to the surface of motor neurons after axotomy.** Representative micrographs of the surface of (**A**,**B**) contralateral and (**C**,**D**) ipsilateral motor neurons from C3H/HePas wild-type (WT) and C3H/HeJ TLR4 mutant mice. At 1 week after peripheral lesion, WT mice had greater preservation of synaptic terminals from the surface of (**A**) unlesioned and (**B**) lesioned motor neurons. (**E**) Graph of the total synaptic contact in unlesioned and lesioned neurons; there was greater synaptic covering in WT mice compared with TLR4 mutant mice on both unlesioned and lesioned sides. The black arrows indicate the location of motor-neuron membrane surfaces from which synaptic terminals were (**C**) in contact with or (**D**) detached from the surface of the motor neuron. The white asterisk indicates a microglial cell close to a synaptic terminal. **E**) Graph of the total synaptic covering in unlesioned and lesioned neurons; there was greater synaptic covering in WT mice compared with TLR4 mutant mice on both sides. (**F**) Graph of the total number of synaptic terminals per 100 μm of motor-neuron membrane; note the difference between genotypes on the lesioned side. (**G**) Graph of the total bouton length on the motor-neuron membrane in lesioned and unlesioned neurons; the difference is presented before axotomy. (**H**–**J**) Graphs of F-, S- and C-terminal numbers per 100 μm of motor-neuron membrane, respectively. A greater preservation of F-type terminals was seen before and after axotomy in WT mice, and, a greater preservation of S-type terminals was seen before lesioning in WT mice. * *P*<0.05 and ** *P*<0.01. Scale bar: 0.5 μm.

In line with the immunostaining data, the absence of TLR4 led to a significantly greater synaptic elimination process (C3H/HePas 45.43% ± 1.15%, C3H/HeJ, 31.19% ± 2.42%, Student *t* test *P*<0.01). Moreover, the unlesioned side of WT animals showed a greater mean bouton length per 100 μm, resulting in an increase of the total synaptic covering compared with the same side of the mutants (C3H/HePas 3.01 ± 0.16; C3H/HeJ 2.56 ± 0.10, Student *t*-test *P*<0.05) (Figure 4G). The ultrastructural results suggest that TLR4 deficiency led to synaptic changes in the mutant animals that were independent of the lesion. However, such changes were not related to the number of terminals, as seen on the lesioned sides (C3H/HePas 48.53 ± 3.56; C3H/HeJ 37.99 ± 1.61, Student *t* test *P*<0.05) (Figure 4F).

The quantitative analysis of the F, S and C boutons was further investigated under TEM (Figure 3H-J). As described by Conradi [21], the presynaptic terminals of motor neurons were typed according to the shape of their synaptic vesicles. On the lesioned side, a greater percentage of F terminals was preserved in apposition to motor neurons in KO mice (C57BL/6J 21.18% ± 1.41%, TLR2^−/−^ 30.62% ± 2.40%, Student *t*-test *P*<0.05) (Figure 3H). In the same context, a greater percentage of F terminals was preserved in apposition to sciatic motor neurons in C3H/HePas mice compared with TLR4 mutant mice (C3H/HePas, 35.99% ± 1.99%, C3H/HeJ 22.36% ± 2.28%, Student *t*-test *P*<0.01) (Figure 4H). Moreover, in WT mice, there was a greater percentage of F terminals on the unlesioned side compared with those in the TLR4 mutants (C3H/HePas 43.60% ± 2.94%, C3H/HeJ 32.38% ± 2.83%; Student *t*-test *P*<0.01) (Figure 4H). As a putative compensatory mechanism in C3H/HePas mice, the proportion of S terminals was also increased in WT mice, possibly to balance the numbers of inhibitory and excitatory terminals (C3H/HePas 11.58% ± 0.95%, C3H/HeJ 6.94% ± 0.62%, Student *t*-test *P*<0.05, Figure 4I). In contrast to the TLR4 mutants, TLR2 KO mice presented a greater proportion of F terminals on the unlesioned side compared with those in WT mice (C57BL/6J 31.74% ± 1.82%, TLR2^−/−^ 42.95% ± 2.90%, Student *t*-test *P*<0.05) (Figure 3H). No differences in the S terminals on the lesioned side of TLR2 or TLR4 mice were seen.

### The absence of Toll-like receptor 2 is associated with the downregulation of pro-inflammatory interleukins and astroglial reactivity marker glial fibrillary acidic protein *in vivo* and *in vitro*

The effects of sciatic nerve transection on astroglial reaction were assessed by quantitative measurements of immunoreactivity around the spinal-cord motor nucleus.

**Figure 5:**
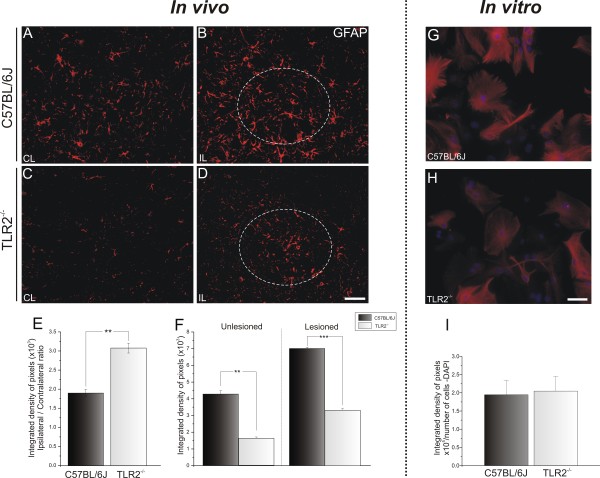
**Representative images of glial fibrillary acidic protein (GFAP) immunostaining in C57BL/6J wild-type (WT) and Toll-like receptor (TLR)2 knockout (KO) mice (TLR2**^**−/−**^**),1 week after unilateral axotomy.** (**A**,**C**) Contralateral side of the lumbar spinal cord; note the increased basal reactivity in WT compared with TLR2^−/−^. (**B**,**D**) The lesion upregulated GFAP labeling in both groups, but there was higher expression in WT mice. (**E**) Graph representing the ipsilateral:contralateral ratio of the integrated density of pixels. (**F**) Graph representing the quantification of the integrated density of pixels on unlesioned and lesioned sides ** *P*<0.01 and *** *P*< 0.001. (**G**,**H**) Purified astrocyte cultures showing astroglial reactivity in WT and TLR2^−/−^ cultures. (**I**) Graph showing no significant difference in glial fibrillary acidic protein (GFAP) immunolabeling between the experimental groups. Scale bar: 50 μm.

**Figure 6:**
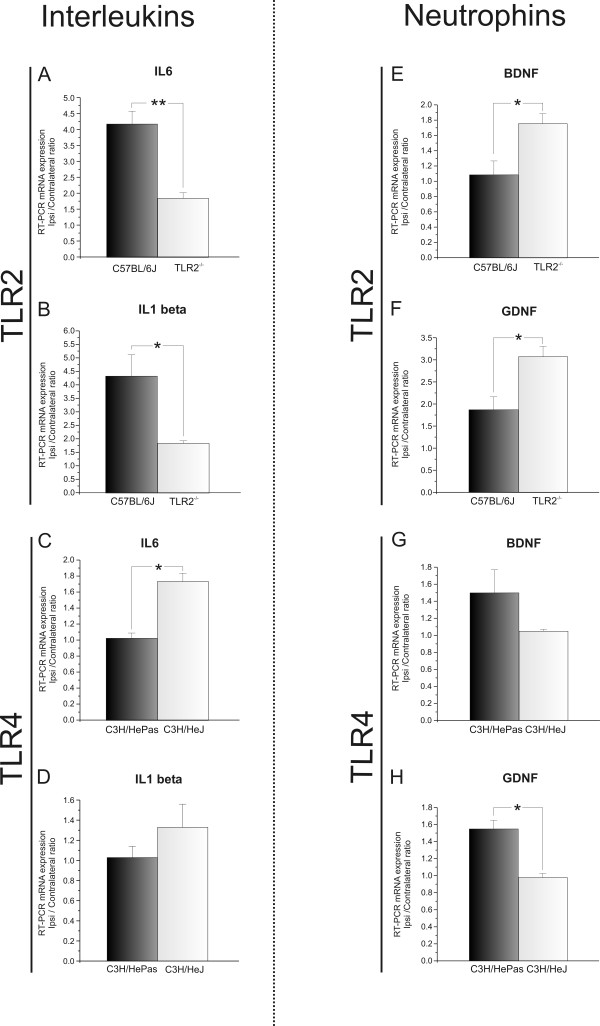
**Interleukin and neurotrophin mRNA levels after axotomy.** (**A**,**B**) Graphs showing interleukin (IL)-6 and IL-1β mRNA expression in the lumbar spinal cord of C57BL/6J wild-type (WT) and Toll-like receptor (TLR)2 knockout (KO) mice (TLR2^−/−^). (**C**,**D**) Graphs showing IL-6 and IL-1β mRNA expression in the lumbar spinal cord of C3H/HePas WT and C3H/HeJ TLR4 mutants. (**E**,**F**) Graphs showing brain-derived neurotrophic factor (BDNF) and glial cell-derived neurotrophic factor (GDNF) mRNA levels in the lumbar spinal cord of C57BL/6J WT and TLR2^−/−^ mice. (**G**,**H**) Graphs showing BDNF and GDNF mRNA levels in the lumbar spinal cord of C3H/HePas WT and C3H/HeJ TLR4 mutants. Note that the absence of TLR2 downregulated IL-6 and IL-1β, and upregulated GDNF and BDNF mRNA expression after unilateral sciatic nerve axotomy, whereas the mutation of TLR4 resulted in upregulation of IL-6 and downregulation of GDNF mRNA. * *P*<0.05Scale Bar: 50 μm.

The basal expression on the unlesioned side was different between WT and TLR2-KO mice (C57BL/6J 4.27 × 10^3^ ± 0.20 × 10^3^; TLR2^−/−^, 1.61 × 10^3^ ± 0.08 × 10^3^, Student *t*-test, *P*<0.01) (Figure 5A,C). Nevertheless, *in vitro* astrocyte cultures displayed similar GFAP immunoreactivity (Figure 5G,H). After lesioning occurred, astroglial reactivity was proportionally upregulated in WT and KO mice (C57BL/6J 7.02 × 10^3^ ± 0.08 × 10^3^ TLR2^−/−^ 3.33 × 10^3^ ± 0.1 × 10^3^, Student *t*-test, *P*<0.001) (Figure 5B,D). These findings suggest that TLR2 signaling interferes with astroglial reactivity even before lesioning. To investigate the possibility of astroglial interference further, the levels of IL-6 and IL-1β mRNAs were quantified by real-time RT-PCR. The absence of TLR2 was associated with decreased expression of pro-inflammatory interleukins in the spinal cord after peripheral axotomy (IL-6: C57BL/6J 4.19 ± 0.40, TLR2^−/−^ 1.86 ± 0.17, Student *t*-test *P*<0.05; IL-1β: C57BL/6J 4.34 ± 0.79, TLR2^−/−^ 1.85 ± 0.09, Student *t*-test *P*<0.05) (Figure 6A,B).

**Figure 7:**
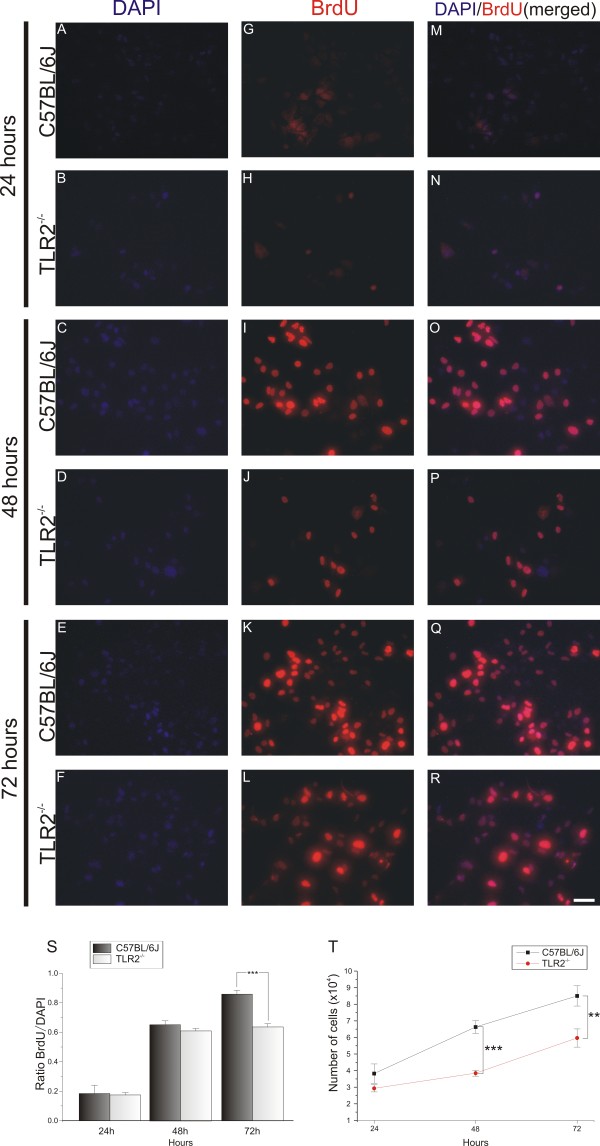
**Cell proliferation assay in purified astrocyte cultures from C57BL/6J wild-type (WT) and Toll-like receptor (TLR)2 knockout (KO) mice (TLR2**^**−/−**^**) mice.** (**A**-**R**) 4^′^,6-diamidino-2-phenylindole (DAPI and 5-bromo-2^′^-deoxyuridine (BrdU) immunostaining after culturing for 3 days. (**A**-**L**) Single labeling with (**A**-**F**) DAPI And (**G**-**L**) BrdU. (**M**-**R**) Double labeling with DAPI and BrdU; note the greater amount of nuclear staining in WT-derived cultures. (**S**) Graph showing the ratio of BrdU:DAPI; note that WT cell cultures displayed a greater proliferation rate, especially at 72 hours. (**T**) Graph showing the astrocyte number for each day; the absence of TLR2 affected the cell growth after 48 hours. ** *P*<0.01; *** *P*<0.001. Scale bar:50 Î¼m.

Astrocyte primary cultures were prepared to investigate *in vitro* astroglial hyperplasia and hypertrophy. An important question to answer was whether, besides the glial hypertrophy, the absence of TLR2 also had some effect on astrocyte proliferation (hyperplasia). For this purpose, DAPI staining of nuclear DNA was used to analyze the number of viable astrocytes in cultures from WT and KO mice. The results indicate a greater number of BrdU-positive cells in the WT cultures compared with the KO-derived cultures (Figure 7G-L). The mitotic rate was calculated from the ratio of BrdU/DAPI labeling. After 24 or 48 hours of culturing, no significant differences were seen between the genotypes, but at 72 hours, the mitotic rate was significantly greater in the control cultures than in their KO counterparts (78 hours: C57BL/6J 0.86 ± 0.27, TLR2^−/−^ 0.63 ± 0.02, two-way ANOVA followed by Bonferroni *post hoc* test *P*<0.001) (Figure 7S). The absence of TLR2 had a significant effect on the astrocyte proliferation rate (Figure 6T). After 24 hours of culturing, the number of astrocytes in the control-derived cultures was significantly greater than in KO-derived cultures. The differences between the WT and KO cultures were significant at 48 and 78 hours (number of DAPI-labeled cells at 48 hours: C57BL/6J 6.62 × 10^4^ ± 0.38 × 10^4^; TLR2^−/−^ 3.84 × 10^4^ ± 0.17 × 10^4^; *P*<0.001; 72 hours: C57BL/6J, 8.5 × 10^4^ ± 0.61 × 10^4^; TLR2^−/−^ 5.9 × 10^4^ ± 0.55 × 10^4^, two-way ANOVA followed by Bonferroni *post hoc* test *P*<0.01).

### Glial reactivity and inflammation-related interleukin mRNA levels in the absence of Toll-like receptor 4 signaling

**Figure 8:**
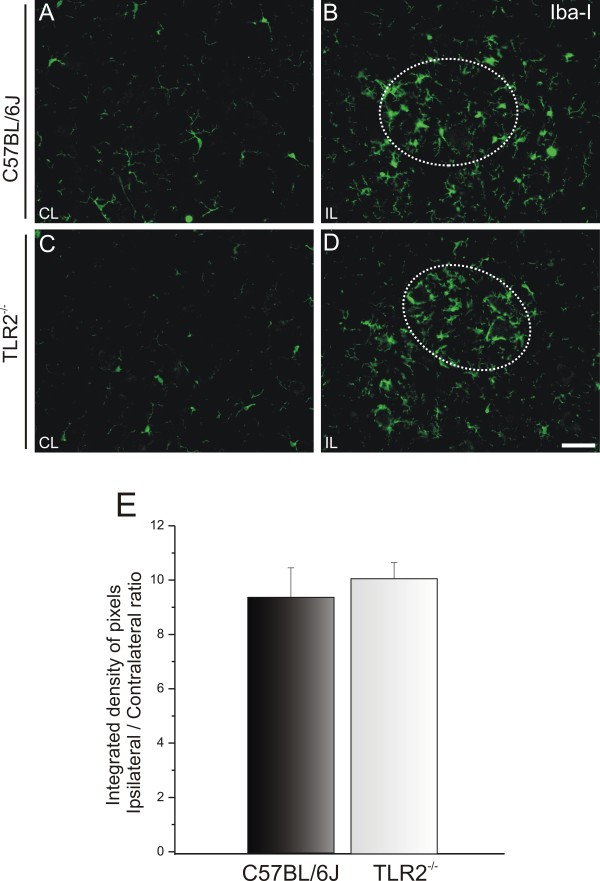
**Representatives images of microglial immunostaining in C57BL/6J wild-type (WT) and Toll-like receptor (TLR)2 knockout (KO) mice (TLR2**^**−/−**^**) mice, 1 week after sciatic nerve unilateral axotomy.** (**A**,**C**) Contralateral side and (**B**,**D**) ipsilateral side of WT and TLR2^−/−^ mice. Iba1 expression increased, particularly on the surface of the axotomized neurons of both groups, but no differences between the groups were detected. The dashed circle indicates the motor nucleus containing the alpha motor neurons. (**E**) Graph representing the ipsilateral:contralateral ratio of the integrated density of pixels (*P*>0.05). Scale bar: 50 μm.

**Figure 9:**
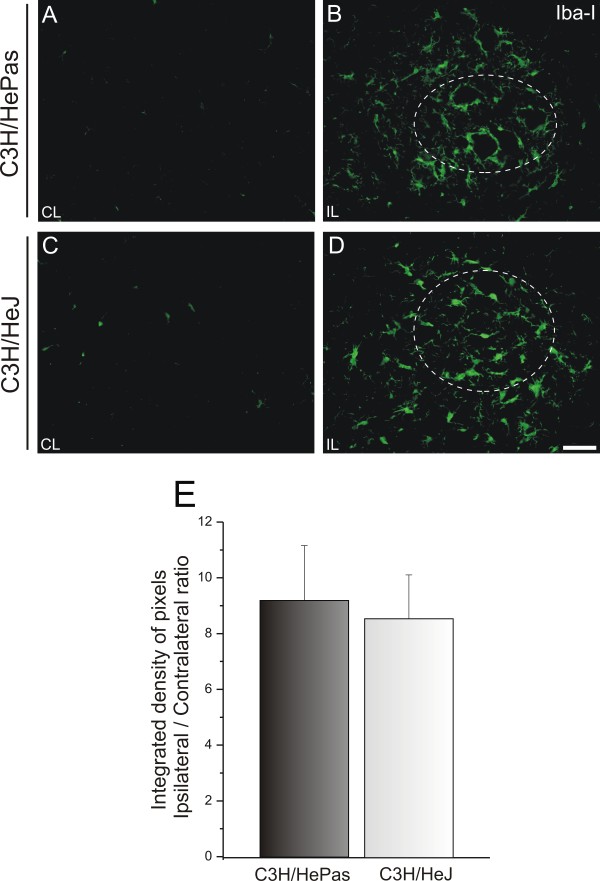
**Representative images of microglial immunostaining in C3H/HePas wild-type (WT) and C3H/HeJ Toll-like receptor (TLR)4 mutant mice 1 week after sciatic nerve unilateral axotomy.** (**A**,**C**) Unlesioned side and (**B**,**D**) lesioned side of the WT and TLR4 mutant mice. Note the higher Iba1 expression on the surface of the axotomized neurons in both groups, with no differences between groups. The dashed circle indicates the motor nucleus containing the alpha motor neurons. (**E**) Graph representing the ipsilateral:contralateral ratio of the integrated density of pixels (*P*>0.05). Scale bar: 50 μm.

The microglial reactivity was negligible in the unlesioned material for all four groups (Figure 8, Figure 9). Additionally, although the axotomy led to increased microglial reactivity, no significant differences of groups could be seen on the lesioned side (ipsilateral/contralateral side ratio: C57BL/6J 9.41 ± 1.09, TLR2^−/−^ 10.12 ± 0.59 (Figure 8); ipsilateral/contralateral side ratio: C3H/HePas 9.20 ± 1.57; C3H/HeJ 8.57 ± 1.97 (Figure 9)).

**Figure 10:**
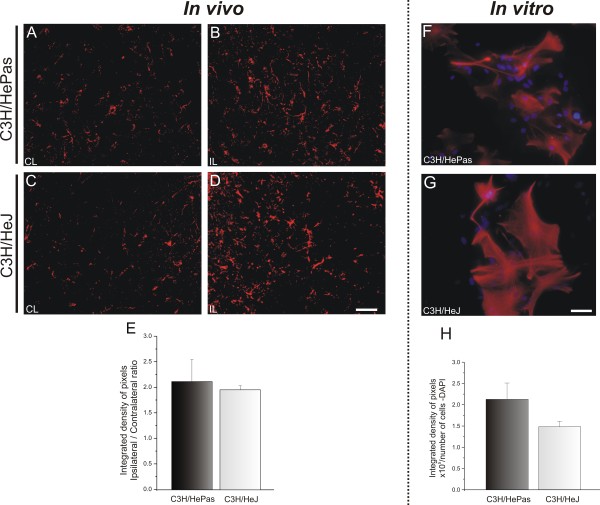
**Representative images of glial fibrillary acidic protein (GFAP) immunostaining in C3H/HePas wild-type (WT) and C3H/HeJ Toll-like receptor (TLR)4 mutant mice 1 week after unilateral axotomy.** (**A**, **C**) Contralateral side and (**A**,**C**) ipsilateral side of the of the lumbar spinal cord of C57BL/6J WT and TLR2^−/−^ mice. The lesion upregulated GFAP labeling in both groups, with no differences between groups. (**E**) Graph representing the ipsilateral:contralateral ratio of the integrated density of pixels. (**F**,**G**) Purified astrocyte cultures showing astroglial reactivity in C3H/HePas WT and C3H/HeJ TLR4 mutant cultures. (**H**) Graph showing no significant difference in GFAP immunolabeling between these two groups. Scale bar: 50 μm.

Regarding astroglial reaction, GFAP labeling was found to be stronger on the ipsilateral side, but no significant differences between TLR4 mutants and WT mice were seen (ipsilateral/contralateral side ratio: C3H/HePas 2.12 ± 0.43, C3H/HeJ 1.96 ± 0.08) (Figure 10E).

The absence of differences between the glial reactions of WT and TLR4 mutant mice was further investigated with RT-PCR to quantify the transcription levels of IL-1β and IL-6. The results show that absence of TLR4 signaling altered IL-6 mRNA expression. However, no changes in IL-1β mRNA expression after axotomy were seen (IL-6: C3H/HePas 1.03 ± 0.064, C3H/HeJ 1.74 ± 0.10, Student *t* test, *P*<0.05; IL-1β: C3H/HePas 1.03 ± 0.11, C3H/HeJ 1.33 ± 0.23) (Figure 6C,D).

Astroglial reactivity was also investigated *in vitro* in astrocyte primary cultures from TLR4-mutant and WT mice. *In vitro* analysis confirmed our *in vivo* results, showing no difference in astrocyte reactivity (C3H/HePas 2.14 × 10^5^ ± 0.38 × 10^5^; C3H/HeJ 1.50 × 10^5^ ± 0.14 × 10^5^) (Figure 10H).

**Figure 11:**
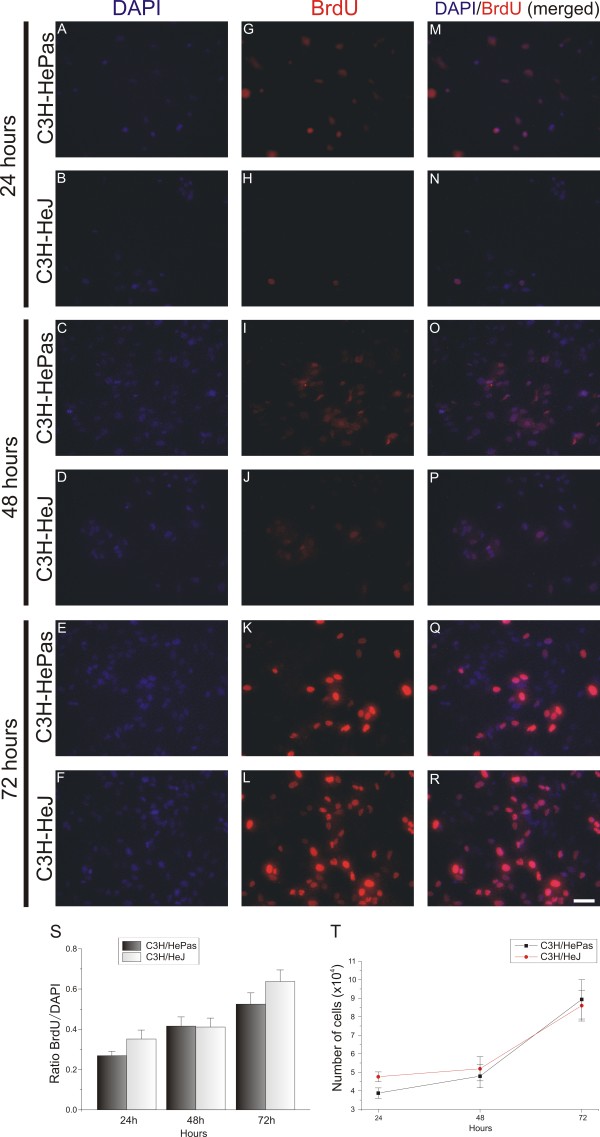
**Cell proliferation assay in purified astrocyte cultures from C3H/HePas wild-type (WT) and C3H/HeJ Toll-like receptor (TLR)4 mutant mice.** (**A**-**R**) 4^′^,6-diamidino-2-phenylindole (DAPI and 5-bromo-2^′^-deoxyuridine (BrdU) immunostaining after culturing for 3 days. (**A**-**L**) Single labeling with (**A**-**F**) DAPI and (**G**-**L**) BrdU. (**M**-**R**) Double labeling with DAPI and BrdU. (S) Graph showing the ratio of BrdU to DAPI; there was no difference in the proliferation rate between WT and TLR4 mutant during 3 days of culturing. (**T**) Graph showing the astrocyte number for each day; TLR4 signaling did not affect astrocyte proliferation. Scale bar: 50 μm.

Next, a cell proliferation assay was carried out using the BrdU incorporation technique. The mitotic rate was analyzed and calculated by the ratio of DAPI to BrdU labeling. No significant differences between the mitotic rates of WT and TLR4 mutant cells were seen (Figure 11). These results indicate that TLR4 deficiency does not interfere with astroglial reactivity or proliferation *in vivo* or *in vitro*.

### Major histocompatibility complex I and neurotrophin mRNA transcription levels in the absence of Toll-like receptor 2 and 4 signaling

**Figure 12:**
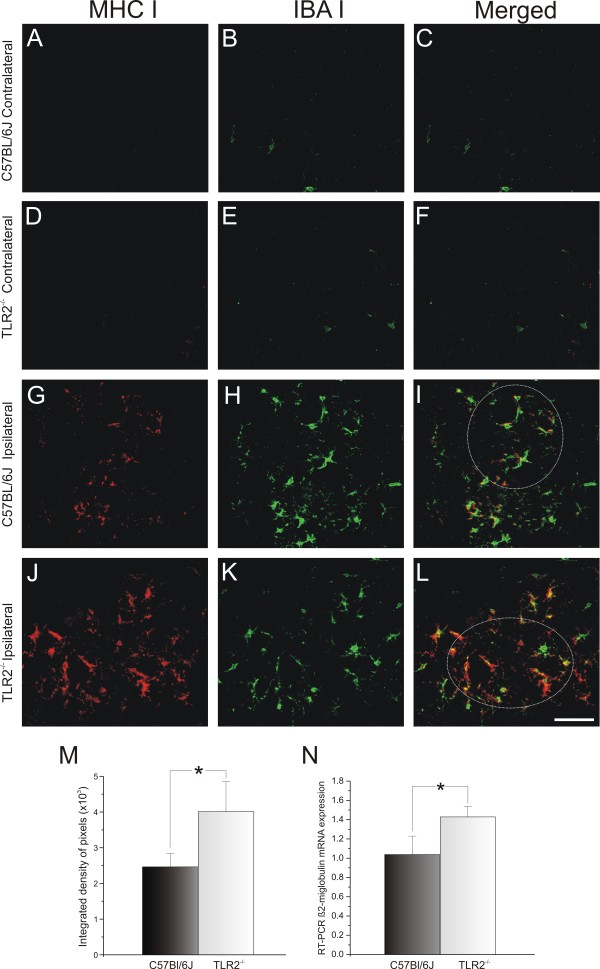
**Major histocompatibility complex class I (MHC I) and Iba1 double staining in C57BL6/J wild-type (WT) and Toll-like receptor (TLR)2 knockout (KO) mice (TLR2**^**−/−**^**) mice 1 week after axotomy.** (**B**,**E**) The contralateral side showed slight IbaI labeling and (**A**,**D**) no MHC I in both groups. There was greater upregulation of MHC I (**J**) in KO mice after lesion compared with (**G**) WT. (**I**,**L**) Colocalization of Iba1 and MHC I labeling, indicating that microglia express MHC I. (**M**) Graph representing the quantification of the integrated density of pixels for MHC I immunolabeling adjacent to motor neurons. (**N**) β2-Microglobulin mRNA level in the lumbar spinal cord determined by reverse transcriptase PCR; both graphs show greater MHC I upregulation in TLR2^−/−^ mice after unilateral sciatic nerve axotomy. * *P*<0.05. Scale bar: 50 μm.

At 1 week after axotomy, a clear change in the MHC class I level was detected in the lesioned motor neurons from C57BL/6J and KO mice. The protein expression was clearly greater on the lesioned side in both strains (compare Figure 12G,J with Figure 12A,D). However, the KO mice showed higher MHC class I expression around the motor neurons compared with the WT (C57BL/6J 2.47 × 10^3^ ± 0.38 × 10^3^; TLR2^−/−^ 4.03 × 10^3^ ± 0.85 × 10^3^, Mann–Whitney *U*-test *P*<0.05) (Figure 12M). To determine whether microglia expressed classic MHC I, double labeling was performed using antibodies against Iba-1 and MHC I (Figure 12).

There was colocalization between the microglial profiles and the MHC I labeling (Figure 12I,L), indicating that the microglia were responsible for the high levels of MHC I expression in KO mice. RT-PCR was performed to examine the transcription levels of β2-microglobulin in the absence of TLR2. The transcription of β2-microglobulin was significantly higher in KO mice (C57BL/6J 1.04 ± 0.21, TLR2^−/−^ 1.43 ± 0.11, Student *t*-test, *P*<0.05) (Figure 12N). These findings show that TLR2 signaling interferes with MHC I expression. Moreover, the absence of TLR2 influenced the upregulation of neurotrophin expression in the CNS; there was higher expression of BDNF and GDNF mRNAs in KO mice (BDNF: C57BL/6J 1.09 ± 0.18, TLR2^−/−^ 1.76 ± 0.13, Student *t*-test *P*<0.05, (Figure 7E); GDNF: C57BL/6J 1.88 ± 0.29, TLR2^−/−^ 3.08 ± 0.23, Student *t*-test *P*<0.05 (Figure 6F).

The possibility that TLR4 signaling modulates neurotrophin expression and enhances synaptic preservation was investigated with RT-PCR. In this way, the transcription levels of BDNF and GDNF was compared between the different mice strains. The results showed that preservation of synaptic covering after lesion in the TLR4 signaling correlated with the upregulation of neurotrophin expression (BDNF: C3H/HePas 1.50 ± 0.27, C3H/HeJ 1.05 ± 0.02, Student *t*-test, *P*>0.05 (Figure 7G); GDNF: C3H/HePas 1.55 ± 0.10, C3H/HeJ 0.98 ± 0.04, Student *t*-test, *P*<0.05 (Figure 6H)).

**Figure 13:**
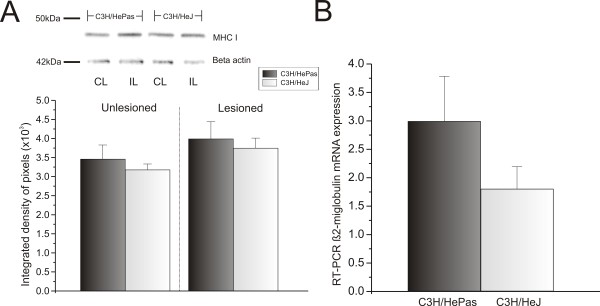
**Major histocompatibility complex class I (MHC I) and β2-microglobulin mRNA levels in the spinal cord of wild-type (WT) and C3H/HeJ Toll-like receptor (TLR)4 mutant mice after axotomy.** (**A**) Western blot analysis of MHC I expression in the lumbar spinal cord of the contralateral and ipsilateral sides. MHC I was upregulated after lesion, with no difference between groups. β-Actin was used as sample loading control. UL = unlesioned; L = lesioned. (**B**) β2-Microglobulin mRNA level determined by reverse transcriptase PCR in the lumbar spinal cord of C3H/HePas WT and C3H/HeJ TLR4 mutant mice. (**B**) Graph showing β2-microglobulin mRNA upregulation after nerve injury, with no difference between groups.

MHC I expression could not be detected by immunolabeling in C3H/HePas and C3H/HeJ because of the properties of the antibody, which recognizes their specific MHC I haplotype. The antibody was thus only suitable for western blotting evaluation. Thus, for the afore mentioned strains, the immunostaining was substituted with western blotting, using protein extracts obtained from the lumbar intumescence after peripheral nerve lesion. MHC I protein was detected, but no significant differences between genotypes were seen (contralateral: C3H/HePas 3.46 × 10^3^ ± 0.37 × 10^3^; C3H/HeJ 3.18 × 10^3^ ± 0.15 × 10^3^; ipsilateral: C3H/HePas 3.99 × 10^3^ ± 0.45 × 10^3^; C3H/HeJ 3.75 × 10^3^ ± 0.26 × 10^3^) (Figure 13A). Similarly, the RT-PCR results did not show significant differences in β2-microglobulin mRNA expression (C3H/HePas 2.99 ± 0.79; C3H/HeJ 1.80 ± 0.39) (Figure 13B).

## Discussion

The retrograde response to axotomy is a well-known phenomenon that takes place in the surroundings of lesioned motor neurons in the spinal cord. This reaction to injury has been extensively investigated, and there is evidence that such changes in the spinal-cord microenvironment may determine the quality of the regenerative outcome. Several molecules have been implicated in this process, including some characteristically related to the classic and non-classic immune responses, including MHC I, T-cell receptor subcomponents, and complement cascade elements. Thus, many molecules that were once thought to be linked exclusively to immune processes were recognized to be present in the CNS and expressed by neurons and glia [23]. TLRs, which are major pattern-recognition receptors of the innate immune system, play an important role in CNS plasticity [24-31]. Therefore, we investigated in the present study the question of whether TLRs might participate in or influence synaptic stability after peripheral lesion.

Our results indicate that TLR2 and TLR4 have opposite effects. We found that the absence of TLR4 signaling leads to a greater loss of synaptic contacts to large motor neurons after distal axonal injury, suggesting a protective role for TLR4 with respect to the spinal-cord circuits.

One hypothesis to explain this result is that TLR4 ligands are present in the pathological CNS and thus could regulate CNS microglia activation [9,10,15]. Reactive glial cells are involved in modulating the synaptic processes, inducing displacement of the presynaptic terminals from axotomized motor neurons [5,18,19]. Additionally, reactive glial cells release ILs within the lesion site [32,33], which might be neuroprotective and influence the upregulation of neurotrophins and other growth factors [34-36]. However, overexpression of ILs can activate inflammatory responses, leading to degeneration and neuronal loss [37]. The results of the present work reveal that TLR4 signaling did not interfere with glial reactivity, but did influence the upregulation of GDNF gene expression and downregulated IL-6. These facts suggest that TLR4 signaling contributes to the preservation of the synaptic contacts in an MHC I-independent manner.

In contrast to the findings related to TLR4 expression, TLR2 signaling was implicated in the reduction of synaptic contacts after peripheral axotomy. However, this reduction in presynaptic terminals might be related to different reasons. As seen in the *in vitro* and *in vivo* experiments, TLR2 expression correlated with increased astroglial proliferation and reactivity. This finding is particularly interesting because increases in astroglial reactivity has been related to greater stripping of the synaptic boutons [6]. Therefore, it is possible that TLR2 expression overcomes the importance of astrogliosis in the synaptic stripping process. As noted previously, both microglial and astroglial cells may be implicated in synaptic elimination after neuronal damage [5,6,18]. Although the microglial reaction in our study did not differ between the two mouse strains, MHC I protein was co-localized with the Iba-1 marker, indicating that microglia upregulated the expression of MHC I proteins soon after axotomy. Furthermore, our data show increased MHC class I gene and protein expression in KO mice. As mentioned previously, MHC I plays an important role in the maintenance of synaptic terminals [2]. Therefore, transgenic mice with enhanced neuronal MHC I expression present a better regenerative outcome [38]. The present results indicate that TLR2 expression surpasses MHC I in importance with respect to synaptic stability because TLR2^−/−^ mice exhibited high MHC I levels but a greater preservation of synaptic inputs. This finding is important as it indicates that the involvement of immune-related molecules is even more complex and far-reaching than previously anticipated.

In addition to the roles discussed above, TLR2 may also influence the secretion of pro-inflammatory cytokines, such as IL-1β, IL-6, tumor necrosis factor (TNF)-α, and chemokines, including interferon-γ, that are released by activated microglia and astrocytes [39]. Astrocytes are the major source of IL-6 in CNS injury [32], partly because TNF-α and IL-1β signaling can upregulate IL-6 production by astrocytes [34]. The absence of TLR2 reduces pro-inflammatory interleukins and, in turn, microglial and astrocyte activation [39]. The present results are consis-tent with this paradigm; KO mice had significantly reduced astroglial reactivity and reduced IL-1β and IL-6 mRNA levels.

Neuroinflammation is involved in the loss of synaptic contacts [40]. However, BDNF promotes survival and axonal regeneration in injured spinal motor neurons [41], modulates synaptic transmission, and regulates the density of synaptic innervation in neurons [42,43]. GDNF is another important trophic factor for motor neurons during axonal regeneration [41,44]. In the present study, BDNF and GDNF mRNAs were both upregulated in TLR2-KO mice 1 week after lesioning. This fact strongly suggests that the absence of TLR2 consistently contributed to the preservation synaptic contacts even though MHC I was significantly upregulated.

In addition to the preservation of inputs, the balance between excitatory and inhibitory inputs is pivotal. This concept was further investigated in the present study by means of TEM. BDNF expression is believed to shift the ratio between excitatory and inhibitory synaptic inputs to the motor neurons towards a dominance of inhibition [43]. As described by Lindå [17], there is preferential elimination of glutamatergic boutons over glycinergic/GABAergic boutons after axotomy. This fact may reflect the existence of a neuroprotective response to injury that helps to avoid excitotoxicity.

Although no difference was detected in glutamatergic terminals, based on our results, there was a prefe-rential preservation of inhibitory terminals, specifically F-terminals, in KO mice after axotomy. Moreover, TLR2 KO mice showed a significant increase in the terminal length that was in contact with the motor neuron membrane, even on the contralateral (unlesioned) side. This finding reinforces the idea that TLR2 signaling could be involved in synaptic stability even before injury, as discussed for TLR4 signaling.

## Conclusion

The present data suggest new, opposing functions for TLR2 and TLR4 in the nervous system. TLR4 may be related to synaptic stability and TLR2 to synaptic elimination after peripheral nerve lesion in adult animals. Our findings not only reinforce the importance of immune molecules for the remodeling of the nervous system after injury but also present new perspectives for putative treatments of neurological disorders that are characterized by synaptic loss.

## Abbreviations

BSA: Bovine serum albumin; DMEM: Dulbecco’s modified Eagle’s medium; EDTA: Ethylene diamene tetraacetic acid; PBS: Phosphate-buffered saline.

## Competing interests

The authors declare that they have no competing interests.

## Authors' contributions

ALRO and LAV provided designed the study, supervision, analyzed the data, and wrote the manuscript. CMF designed and performed the experiments, analyzed the data, prepared the figures, and wrote the manuscript. All authors read and approved the final manuscript.

## Supplementary Material

Additional file 1: Figure S1 Summary of the immunolabeling quantification procedures carried out using the Image J software. **(A)** The same image is opened twice and put side to side. **(B)** Threshold is obtained by comparison with the original image, so that the immunolabeling is highlighted. **(C)** Measurement procedure around a large motor neuron present in the dorsolateral lamina IX. **(D)** Schematic view of the spinal cord showing the sciatic nerve pool of motor neurons used for the measurements. One motor neuron within the group is enlarged in order to illustrate the immunolabeled sampled areas. click here for file

## References

[B1] Huh GS Boulanger LM Du H Riquelme PA Brotz TM Shatz CJ Functional requirement for class I MHC in CNS development and plasticity Science 2000 290 5499 2155 2159 10.1126/science.290.5499.2155 11118151 PMC2175035

[B2] Oliveira AL Thams S Lidman O Piehl F Hökfelt T Kärre K Lindå H Culheim S A role for MHC class I molecules in synaptic plasticity and regeneration of neurons after axotomy Proc Natl Acad Sci 2004 101 17843 17848 10.1073/pnas.0408154101 15591351 PMC539738

[B3] Berg A Zelano J Stephan A Thams S Barres BA Pekny M Pekna M Cullheim S Reduced removal of synaptic terminals from axotomized spinal motoneurons in the absence of complement C3 Exp Neurol 2012 237 1 8 17 10.1016/j.expneurol.2012.06.008 22721768

[B4] Fourgeaud L Boulanger LM Synapse remodeling, compliments of the complement system Cell 2007 131 6 1034 1036 10.1016/j.cell.2007.11.031 18083091

[B5] Aldskogius H Liu L Svensson M Glial responses to synaptic damage and plasticity J Neurosci Res 1999 58 1 33 41 10.1002/(SICI)1097-4547(19991001)58:1<33::AID-JNR5>3.0.CO;2-M 10491570

[B6] Emirandetti A Zanon RG Sabha JR Oliveira ALR Astrocyte reactivity influences the number of presynaptic terminals apposed to spinal motoneurons after axotomy Brain Res 2006 1095 35 42 10.1016/j.brainres.2006.04.021 16714003

[B7] Lehnardt S Lachance C Patrizi S Lefebvre S Follett PL Jensen FE Rosenberg PA Volpe JJ Vartanian T The toll-like receptor TLR4 is necessary for lipopolysaccharide-induced oligodendrocyte injury in the CNS J Neurosci 2002 22 7 2478 2486 11923412 10.1523/JNEUROSCI.22-07-02478.2002 PMC6758325

[B8] Lehnardt S Massillon L Follett P Jensen FE Ratan R Rosenberg PA Volpe JJ Vartanian T Activation of innate immunity in the CNS triggers neurodegeneration through a Toll-like receptor 4-dependent pathway Proc Natl Acad Sci U S A 2003 100 14 8514 8519 10.1073/pnas.1432609100 12824464 PMC166260

[B9] Lehnardt S Innate immunity and neuroinflammation in the CNS: the role of microglia in Toll-like receptor-mediated neuronal injury Glia 2010 58 3 253 263 19705460 10.1002/glia.20928

[B10] Jack CS Arbour N Manusow J Montgrain V Blain M McCrea E Shapiro A Antel JP TLR signaling tailors innate immune responses in human microglia and astrocytes J Immunol 2005 22 4320 4330 16177072 10.4049/jimmunol.175.7.4320

[B11] Henn A Kirner S Leist M TLR2 hypersensitivity of astrocytes as functional consequence of previous inflammatory episodes J Immunol 2011 186 5 3237 3247 10.4049/jimmunol.1002787 21282508

[B12] Goethals S Ydens E Timmerman V Janssens S Toll-like receptor expression in the peripheral nerve Glia 2010 58 14 1701 1709 10.1002/glia.21041 20578041

[B13] Tang SC Arumugam TV Xu X Cheng A Mughal MR Jo DG Lathia JD Siler DA Chigurupati S Ouyang X Magnus T Camandola S Mattson MP Pivotal role for neuronal Toll-like receptors in ischemic brain injury and functional deficits Proc Natl Acad Sci USA 2007 104 34 13798 13803 10.1073/pnas.0702553104 17693552 PMC1959462

[B14] Bowman CC Rasley A Tranguch SL Marriott I Cultured astrocytes express toll-like receptors for bacterial products Glia 2003 43 3 281 291 10.1002/glia.10256 12898707

[B15] Olson JK Miller SD Microglia initiate central nervous system innate and adaptive immune responses through multiple TLRs J Immunol 2004 173 6 3916 3924 15356140 10.4049/jimmunol.173.6.3916

[B16] Phulwani NK Esen N Syed MM Kielian T TLR2 expression in astrocytes is induced by TNF-alpha- and NF-kappa B-dependent pathways J Immunol 2008 181 6 3841 3849 18768838 10.4049/jimmunol.181.6.3841 PMC2649826

[B17] Lindå H Shupliakov O Örnung G Ottersen OP Storm-Mathisen J Risling M Cullheim S Ultrastructural evidence for a preferential elimination of glutamate-immunoreactive synaptic terminals from spinal motoneurons after intramedullary axotomy J Comp Neurol 2000 425 10 23 10.1002/1096-9861(20000911)425:1<10::AID-CNE2>3.0.CO;2-# 10940938

[B18] Cullheim S Thams S The microglial networks of brain and their role in neural network plasticity after lesion Brain Res Rev 2007 55 89 96 10.1016/j.brainresrev.2007.03.012 17509690

[B19] Schiefer J Kampe K Dodt HU Zieglgänsberger W Kreutzberg GW Microglial motility in the rat facial nucleus following peripheral axotomy J Neurocytol 1999 28 6 439 453 10.1023/A:1007048903862 10767097

[B20] Poltorak A He X Smirnova I Liu MY Van Huffel C Du X Birdwell D Alejos E Silva M Galanos C Freudenberg M Ricciardi-Castagnoli P Layton B Beutler B Defective LPS signaling in C3H/HeJ and C57BL/10ScCr mice: mutations in Tlr4 gene Science 1998 282 5396 2085 2088 9851930 10.1126/science.282.5396.2085

[B21] Conradi S On motoneuron synaptology in adult cats Acta Physiol Scand 1969 332 1 57

[B22] McCarthy KD Vellis J Preparation of separate astroglial and oligodendroglial cell cultures from rat cerebral tissue J Cell Biol 1980 85 890 902 10.1083/jcb.85.3.890 6248568 PMC2111442

[B23] Boulanger LM Huh GS Shatz CJ Neuronal plasticity and cellular immunity: shared molecular mechanisms Curr Opin Neurobiol 2001 11 5 568 578 10.1016/S0959-4388(00)00251-8 11595490

[B24] Ma Y Li J Chiu I Wang Y Sloane JA Lü J Kosaras B Sidman RL Volpe JJ Vartanian T Toll-like receptor 8 functions as a negative regulator of neurite outgrowth and inducer of neuronal apoptosis J Cell Biol 2006 175 2 209 215 10.1083/jcb.200606016 17060494 PMC2064562

[B25] Li Y Li H Zhang Y Sun X Hanley GA LeSage G Zhang Y Sun S Peng Y Yin D Toll-like receptor 2 is required for opioids-induced neuronal apoptosis Biochem Biophys Res Commun 2010 391 1 426 430 10.1016/j.bbrc.2009.11.074 19914204 PMC2812588

[B26] Rolls A Shechter R London A Ziv Y Ronen A Levy R Schwartz M Toll-like receptors modulate adult hippocampal neurogenesis Nat Cell Biol 2007 9 9 1081 1088 10.1038/ncb1629 17704767

[B27] Wainwright DA Xin J Mesnard NA Sanders VM Jones KJ Toll-like receptor 2 and facial motoneuron survival after facial nerve axotomy Neurosci Lett 2010 471 1 10 14 10.1016/j.neulet.2009.12.076 20056129 PMC2825370

[B28] Ziegler G Harhausen D Schepers C Hoffmann O Röhr C Prinz V König J Lehrach H Nietfeld W Trendelenburg G TLR2 has a detrimental role in mouse transient focal cerebral ischemia Biochem Biophys Res Commun 2007 359 3 574 579 10.1016/j.bbrc.2007.05.157 17548055

[B29] Okun E Griffioen KJ Mattson MP Toll-like receptor signaling in neural plasticity and disease TINS 2011 34 5 269 281 21419501 10.1016/j.tins.2011.02.005 PMC3095763

[B30] Kigerl KA Lai W Rivest S Hart RP Satoskar AR Popovich PG Toll-like receptor (TLR)-2 and TLR-4 regulate inflammation, gliosis, and myelin sparing after spinal cord injury J Neurochem 2007 102 1 37 50 10.1111/j.1471-4159.2007.04524.x 17403033

[B31] Boivin A Pineau I Barrette B Filali M Vallieres N Rivest S Lacroix S Toll like receptor signaling is critical for wallerian degeneration and functional recovery after peripheral nerve injury J Neurosci 2007 27 12565 12576 10.1523/JNEUROSCI.3027-07.2007 18003835 PMC6673340

[B32] Benveniste EN Sparacio SM Norris JG Grenett HE Fuller GM Induction and regulation of interleukin-6 gene expression in rat astrocytes J Neuroimmunol 1990 30 2–3 201 212 2121800 10.1016/0165-5728(90)90104-u

[B33] Lee SC Liu W Dickson DW Brosnan CF Berman JW Cytokine production by human fetal microglia and astrocytes. Differential induction by lipopolysaccharide and IL-1 beta J Immunol 1993 150 7 2659 2667 8454848

[B34] John GR Lee SC Song X Rivieccio M Brosnan CF IL-1-regulated responses in astrocytes: relevance to injury and recovery Glia 2005 49 2 161 176 10.1002/glia.20109 15472994

[B35] Herx LM Rivest S Yong VW Central nervous system-initiated inflammation and neurotrophism in trauma: IL-1 beta is required for the production of ciliary neurotrophic factor J Immunol 2000 165 4 2232 2239 10925311 10.4049/jimmunol.165.4.2232

[B36] Pinteaux E Rothwell NJ Boutin H Neuroprotective actions of endogenous interleukin-1 receptor antagonist (IL-1ra) are mediated by glia Glia 2006 53 5 551 556 10.1002/glia.20308 16374779

[B37] Hailer NP Vogt C Korf HW Dehghani F Interleukin-1beta exacerbates and interleukin-1 receptor antagonist attenuates neuronal injury and microglial activation after excitotoxic damage in organotypic hippocampal slice cultures Eur J Neurosci 2005 21 9 2347 2360 10.1111/j.1460-9568.2005.04067.x 15932594

[B38] Joseph MS Bilousova T Zdunowski S Wu ZP Middleton B Boudzinskaia M Wong B Ali N Zhong H Yong J Washburn L Escande-Beillard N Dang H Edgerton VR Tillakaratne NJ Kaufman DL Transgenic mice with enhanced neuronal major histocompatibility complex class I expression recover locomotor function better after spinal cord injury J Neurosci 2011 89 3 365 372 10.1002/jnr.22557 PMC3087178 21259323

[B39] Kim D Kim MA Cho IH Kim MS Lee S Jo EK Choi SY Park K Kim JS Akira S Na HS Oh SB Lee SJ A critical role of toll-like receptor 2 in nerve injury-induced spinal cord glial cell activation and pain hypersensitivity J Biol Chem 2007 282 20 14975 14983 10.1074/jbc.M607277200 17355971

[B40] Rao JS Kellom M Kim HW Rapoport SI Reese EA Neuroinflammation and synaptic loss Neurochem Res 2012 37 5 903 910 10.1007/s11064-012-0708-2 22311128 PMC3478877

[B41] Boyd JG Gordon T Glial cell line-derived neurotrophic factor and brain-derived neurotrophic factor sustain the axonal regeneration of chronically axotomized motoneurons in vivo Exp Neurol 2003 183 610 619 10.1016/S0014-4886(03)00183-3 14552902

[B42] Black IB Trophic regulation of synaptic plasticity J Neurobiol 1999 41 1 108 118 10.1002/(SICI)1097-4695(199910)41:1<108::AID-NEU14>3.0.CO;2-U 10504198

[B43] Novikov LN Novikova LN Holmberg P Kellerth J Exogenous brain-derived neurotrophic factor regulates the synaptic composition of axonally lesioned and normal adult rat motoneurons Neuroscience 2000 100 1 171 181 10.1016/S0306-4522(00)00256-6 10996467

[B44] Naveilhan P ElShamy WM Ernfors P Differential regulation of mRNAs for GDNF and its receptors Ret and GDNFR alpha after sciatic nerve lesion in the mouse Eur J Neurosci 1997 9 7 1450 1460 10.1111/j.1460-9568.1997.tb01499.x 9240402

